# Trends and recent progresses of selenium nanoparticles as novel autophagy regulators for therapeutic development

**DOI:** 10.3389/fnut.2023.1116051

**Published:** 2023-02-02

**Authors:** Dongsheng Chen, Hongmei Lu, Yuhe Ma, Yuhe Huang, Tangxin Zhang, Shuhao Fan, Wensen Lin, Yifan Huang, Hua Jin, Yongdui Ruan, Jun-Fa Xu, Jiang Pi

**Affiliations:** ^1^Guangdong Provincial Key Laboratory of Medical Molecular Diagnostics, The First Dongguan Affiliated Hospital, Guangdong Medical University, Dongguan, China; ^2^Institute of Laboratory Medicine, School of Medical Technology, Guangdong Medical University, Dongguan, China; ^3^Dongguan Key Laboratory of Environmental Medicine, School of Public Health, Guangdong Medical University, Dongguan, China

**Keywords:** selenium, selenium nanoparticles, autophagy, regulators, therapeutics

## Abstract

Autophagy, one of the major intracellular degradation systems, plays an important role in maintaining normal cellular physiological functions and protecting organisms from different diseases. Selenium (Se), an essential trace element, is involved in many metabolic regulatory signaling events and plays a key role in human health. In recent years, selenium nanoparticles (Se NPs) have attracted increasing attentions in biomedical field due to their low toxicity, high bioavailability and high bioactivity. Taking the advantage of their advanced biological activities, Se NPs can be used alone as potential therapeutic agents, or combine with other agents and served as carriers for the development of novel therapeutics. More interestingly, Se NPs have been widely reported to affect autophagy signaling, which therefor allow Se NPs to be used as potential therapeutic agents against different diseases. Here, this review suggested the relationships between Se and autophagy, followed by the trends and recent progresses of Se NPs for autophagy regulation in different diseased conditions. More importantly, this work discussed the roles and potential mechanisms of Se NPs in autophagy regulating, which might enhance our understanding about how Se NPs regulate autophagy for potential disease treatment. This work is expected to promote the potential application of Se NPs as novel autophagy regulators, which might benefit the development of novel autophagy associated therapeutics.

## Introduction

Autophagy, an essential and conserved catabolic process, is one of the major intracellular degradation systems that delivers cytoplasmic or invasive materials into autolysosomes for degradation (1). Although there are three typical classes of autophagy (macroautophagy, microautophagy, and chaperone-mediated autophagy), macroautophagy is thought to be the major type of autophagy and is always referred to as “autophagy” (1). Autophagy begins with an isolation membrane, also known as a phagophore to sequester the cargos, such as proteins, organelles, ribosomes and pathogens, in a double-membrane autophagosome (2). Autophagosome matures through fusion with the lysosome to form autolysosomes, which promotes the degradation of the cargos. Lysosomal permeases and transporters could export amino acids and other by-products of degradation back out to the cytoplasm, where they can be re-used for building macromolecules and for the metabolic uses (2). Thus, autophagy is considered to be a cellular recycling factor that promotes energy efficiency and re-uses the non-functional proteins and organelles (3). Dysfunction of autophagy could induce ubiquitination inhibition, reactive oxygen species (ROS) accumulation, mitochondrial disruption and genomic instability, thus leading to lots of diseased conditions, such as cancer, neurodegeneration, infection, and aging (4, 5). And increasing evidences are suggesting that autophagy can serve an adaptive role to protect organisms against different pathologies, which therefore highlights autophagy as a critical signaling event for novel therapeutic strategy development (6, 7).

Selenium (Se), an essential trace element, has been linked to many health benefits in humans and other mammals, such as decreasing the incidence of cancer (8), protecting against cardiovascular diseases (9), treating particular muscle disorders (10), delaying the onset of AIDS in HIV-positive patients and boosting immune function in mammalian development (11, 12). The biological functions of the micronutrient selenium are mediated in large part by selenoproteins, which contain selenocysteine (Sec) in their active site. Glutathione peroxidase 1 (GPx1), the most abundant selenoprotein in mammals, has been considered as one of the major antioxidant enzymes to protect cells from oxidative damage by degrading toxic H_2_O_2_ (13). The iodothyronine deiodinase family of selenoproteins is involved in the regulation of thyroid hormone activity by reductive deiodination (14, 15). Another kind of selenoproteins-thioredoxin reductases (TRs) are involved in the control of antioxidant defense and regulation of transcription factors (15), which therefore play critical roles in cancer development and treatment (16, 17). Selenophosphate synthetase is a selenoenzyme that catalyzes the ATP-dependent synthesis of selenophosphate (12). Methionine-R-sulfoxide Reductase B1 (MsrB1), a zinc-containing selenoprotein, can reduce oxidized methionine residues to repair oxidatively damaged proteins (18). These important functions of selenoproteins, especially for their regulation effects in cellular oxidative stress, highlight the important physiological roles of Se in human health and diseased conditions.

Cell death is an irreversible cessation of life phenomena and the end of life. According to the definition of morphological criteria, cell death is always divided into apoptosis, necrosis, autophagy, ferroptosis, and cell scorching, etc. (19). Se is an essential trace element that also plays crucial role in cell death. Selenium deficiency has been widely reported to result in the dysfunction of cellular metabolism, which would lead to different modes of cell death (20, 21). Selenoproteins have also been proved to regulate autophagy in different conditions, which therefore can be served as a kind of protective agent to inhibit dysfunctioned autophagy (22, 23). Moreover, selenium compounds showed strong ability to promote cell autophagy of infected cells to kill the intracellular pathogens (24, 25), which indicated the potential of selenium compounds to serve as anti-infectious agents by promoting autophagy. Selenium compounds also possessed chemotherapeutic effects against multiple malignant cancers by regulating autophagy (26), which highlighted selenium compounds as a new generation of anticancer agents by regulating autophagy.

Nanotechnology has been developed rapidly in the past few decades, which brings novel possibilities for disease treatment. Selenium nanoparticles (Se NPs) have attracted extensive attentions for the application of selenium-based products due to their advantages, including higher safety, lower toxicity, higher bioavailability and stronger free radical scavenging ability and antioxidant activities compared to selenium element (27–30). Zhang et al. (31) reported that encapsulation of selenium in chitosan nanoparticles could improve selenium availability and protect cells from selenium-induced DNA damage response, which indicated the higher safety, lower toxicity, higher bioavailability of Se NPs compared the organic and inorganic selenium compounds. Chen et al. (30) demonstrated the higher bioavailability of Se NPs, and moreover, they also found that Se NPs showed stronger antioxidant activities compared with selenium compounds. Additionally, Se NPs have also been reported to show similar or enhanced activities against diseased cells than Se compounds, but show reduced cytotoxicity against normal cells (32).

The above advantages thus allow Se NPs to be used as a kind of novel nanosystems or selenium products for disease treatment in different conditions. Firstly, Se NPs can also be used as carriers of drugs or other biomolecules for targeted delivery. By encapsulating drugs/biomolecules into Se NPs, the obtained nanosystem could lead to the increased stability and prolonged circulation (33), which therefore provide the enhanced efficiency with better targeting effects. And Se NPs can be easily designed to suit their needs in terms of size, surface charge, genetic, drug loading and controlled release, which is significant for the construction of novel nanomedicines (34–36). Secondly, Se NPs have excellent antibacterial activity and anti-viral activity, which therefore can be used as anti-infectious agents (37, 38). Thirdly, Se NPs also have superior antitumor activities beyond the conventional inorganic and organic selenium compounds, which allows Se NPs to kill tumor cells directly or enhance the anti-cancer activity of current chemotherapeutics (33, 39). Moreover, Se NPs have also been reported to show immunological regulation effects in immune cells (40). And the regulation of autophagy was closely associated with these biological activities for Se NPs. Increasing evidences are suggesting that Se NPs play an important role in the treatment of cancer and infectious diseases through the regulation of autophagy for more effective tumor cell killing and pathogen clearance.

Due to the critical roles of Se NPs in autophagy regulation, this review summarized the effects and mechanisms of autophagy regulated by Se and Se NPs, especially the recent progresses of Se NPs in the treatment of different diseases by regulating autophagy, which is expected to promote the development of novel therapeutics.

## Association between selenium and autophagy

**FIGURE 1:**
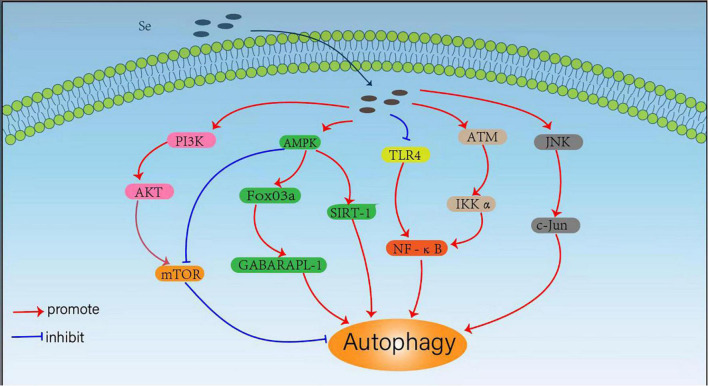
Mechanisms for selenium (Se) regulated cellular autophagy.

The trace element Se can regulate cellular autophagy through different signaling pathways, which is essential for human health. By regulating autophagy under different conditions, selenium compounds and selenoproteins exhibit important effects to induce cancer cell death, reduce drug toxicity, regulate inflammatory responses and resist pathogenic bacterial infections. Considering the critical roles of Se in autophagy regulation, we summarized the mechanisms Se regulates cellular autophagy through various signaling pathways (Figure 1).

Mammalian rapamycin (mTOR) kinase is a well-known negative central regulator of the autophagic process, and its upstream regulatory signals are phosphatidylinositol 3-kinase (PI3K)/protein kinase B (AKT) pathway, which can activate mTOR signalings. The inhibition of PI3K/AKT/mTOR pathway activates autophagy, whereas AMPK downregulates mTOR expression and promotes autophagy by inhibiting the mTOR pathway (41). Qian et al. (22) observed that SeMet could attenuate OTA-induced PCV2 replication by inhibiting autophagy through activation of the PI3K/AKT/mTOR pathway. Cheng et al. (23) also found that L-selenomethionine could inhibit ammonia-induced cardiac autophagy through activation of the PI3K/AKT/mTOR signaling pathway. Yang et al. (42) also found that selenium could inhibit autophagy by regulating the PI3K/AKT/mTOR signaling pathway to prevent ischemia/reperfusion injury-induced blood-brain barrier damage in hyperglycemic patients. It has also been shown that SeMet can upregulate AMPK expression and initiate autophagy to clear tau in 3xTg-AD mice by activating the AMPK/mTOR pathway, suggesting that SeMet might be an effective drug candidate for the treatment of Alzheimer’s disease (AD) (43). Se-Allylselenocysteine (ASC) can also activate autophagy and induce rectal cancer cell death by regulating AMPK/mTOR pathway, which facilitates the development of new therapeutic strategies against colorectal cancer (44).

Another downstream signaling pathway of AMPK is FoxO3a/GABARAPL1, and the activation of AMPK/Fo xO3a/GABARAPL1 pathway can promote cellular autophagy. Yu et al. (26) found that sodium selenite could promote ROS/AMPK/FoxO3a/GABARAPL-1-mediated autophagy to downregulate apoptosis in colon cancer cells and colon xenograft models. In addition, Pant et al. (45) also found that probiotics enriched with selenium could improve hepatic steatosis by regulating AMPK/SIRT-1 mediated autophagy to alleviate NAFLD. The transcription factor NF-κB (nuclear factor kappa B) is not only a regulator of inflammatory response, but also plays an important role in the regulation of autophagy (46). It has been shown that selenium deficiency can induce chicken French bursa autophagy through the ChTLR4/MyD88/NF-κB pathway (47), and sodium selenite can also block TLR4/NF-κB and mitochondrial signaling pathways to inhibit mitochondrial autophagy in bovine support cells exposed to microcystins leucine arginine (MC-LR) (48). Selenite can also regulate the NF-κB signaling pathway by activating the ATM/IKK alpha axis to promote autophagy in leukemic Jurkat cells (49). Moreover, selenium donors can also induce macrophage autophagy through c-Jun-mediated pathway, thereby upregulating c-Jun expression and limiting the intracellular growth of Mycobacterium tuberculosis (25).

Autophagy is an important physiological process that delivers cytoplasmic or invasive materials into autolysosomes for degradation, which is critical to maintain the normal metabolism and thus beneficial to human health. However, in some pathological conditions, abnormal regulations of autophagy may also be harmful to human health. Se has exhibited powerful protective effects on the organism through its dual-role regulation effects on autophagy. It has been shown that maternal Se supplementation during gestation increases the levels of antioxidant selenoproteins and reduces autophagy and inflammation levels in the offspring, which helps to improve the immune function of the offspring (50), whereas maternal Se deficiency induces the dysfunction of autophagy and the damages of placenta (51). Se can also mitigate the damage caused by toxic substances such as cadmium (52–54), lead (55, 56), mercury (57), and ammonia (23, 58) through the inhibition of these toxins-induced and oxidative stress-mediated autophagy. In addition, Se may also attenuate oxidative stress, and ochratoxin A-induced PCV2 replication promotion by inhibiting autophagy (22, 59), which may be beneficial in developing new ideas for the prevention of PCV2 infection. These results indicated that Se can block unhealthy autophagic pathways to protect human health. It has also been shown that Se can exert antitumor effects by promoting autophagy in different tumor cells for more effective tumor inhibition. Sodium selenite can induce autophagy not only in A549 human lung cancer cells by promoting ROS production (60), but also in colorectal cancer cells (26, 61), and these findings could help explore sodium selenite as a potential anti-cancer agent in clinical practice. In addition, Se not only limits the proliferation of Staphylococcus aureus and Mycobacterium tuberculosis by promoting macrophage autophagy (24, 25) but also alleviates the *E. coli*-induced inflammatory response by enhancing bovine mammary epithelial cell autophagy (62), which exhibits potent anti-infective activity. These results suggest that Se can not only block unhealthy autophagic pathways to protect human health, but can also promote healthy autophagic pathways to protect human health.

## Mechanisms for Se NPs regulated autophagy in tumor

Similar with the conventional inorganic or organic selenium compounds, Se NPs can also modulate cellular autophagy through various signaling pathways. Despite being a promising strategy to inhibit cancer progression, current chemotherapy strategies for cancer are still limited due to adverse side effects and poor survival rates. Thus, the development of novel strategies, such as drug-delivery platforms with good biocompatibility that could enhance chemotherapy efficiency and reduce the side effects against cancer, remain a challenge. Wang et al. (63) developed a novel selenium-nanoparticle based drug-delivery agent for cancer treatment from *Kaempferia parviflora* (black ginger) root extract and selenium salts. The obtained KP-Se NPs showed significant cytotoxicity in human gastric adenocarcinoma cells (AGS cells), but showed no significant cytotoxicity in normal cells. These specific anticancer effects of KP-Se NPs were closely related to apoptosis, which was associated with the upregulation of intrinsic apoptotic signaling markers, such as Bcl-2, Bax and caspase 3 in AGS cells. Moreover, KP-Se NPs also caused autophagy of AGS by increasing the autophagic flux-marker protein, LC3B-II, whilst inhibiting autophagic cargo protein, p62. Additionally, phosphorylation of PI3K/Akt/mTOR pathway markers and downstream targets were decreased in KP-SeNP-treated AGS cells. Therefore, this work strongly suggested that KP-Se NPs could act as a novel potential therapeutic agent for GC by regulating autophagy, and also indicated that plant-based synthesis of Se NPs could be considered as one of the best strategies for cancer treatment.

Silymarin is well-known as a traditional hepatoprotective agent, but increasing evidence and clinical results have established the chemopreventive roles of silymarin as a therapeutic option for gastric cancer treatment (64, 65). However, silymarin has major limitations in clinical cancer therapy due to its poor water and lipid solubility (66). Therefore, Mi et al. (67) developed silymarin selenium nanoparticles (Si-Se NPs) by silymarin-mediated green synthesis and investigated their possibility as an anticancer agent. Compared with silymarin, the Si-Se NPs exhibited significantly increased cytotoxic effect of AGS cells without exhibiting toxicity on normal cells. Here, Se NPs could act as a carrier of silymarin to enhance the biocompatibility of silymarin, and silymarin could also further increase the anticancer effects of Se NPs. These Si-Se NPs were also proved to induce autophagy in AGS cells through the inhibition of PI3K/Akt/mTOR pathway. Their results demonstrated that Si-Se NPs could induce stronger inhibition effects on the phosphorylation of PI3Kand Akt than that of silymarin in gastric adenocarcinoma cells, which indicated that the loading of silymarin onto Se NPs could further enhance the anticancer effects of silymarin by inhibiting PI3K/Akt/mTOR pathway. These results demonstrated that the anti-cancer activities of Se NPs are closely associated with their ability to promote cancer cell autophagy by inhibiting PI3K/Akt/mTOR pathway, which could be combined with some anticancer agents, such as silymarin, for more effective PI3K/Akt/mTOR pathway inhibition and autophagy induction in cancer cells.

It is worth noting that the reports of organic molecules composing diselenide-containing nanoparticles can also be applied as autophagy regulators. diselenide-containing fluorescent molecules (SeBDP) and antitumor drug paclitaxel (SePTX) were synthesized and used for constructing SeBDP nanoparticles (SeBDP NPs) and SePTX NPs in aqueous solution through nanoprecipitation method (68). The cellular proliferation inhibition toward tumor cells (including HeLa and MCF-7 cells) was obviously higher than that toward normal cells (BEAS-2B and L929 cells), which might be attributed to the increasing reactive oxygen species in cancer cells treated by Selenium containing nanoparticles. Moreover, they further found that SePTX NPs can successfully induce oxidative stress, cause mitochondrial dysfunction, resulting in mitochondrial pathway-mediated apoptosis, which is related to the upregulation of autophagy-related protein LC3-II (69). These data suggested that SePTX NPs exhibited high inhibiting efficiency against the growth of tumors and were able to reduce the side effects by enhancing cancer cell autophagy.

Se and curcumin have both showed excellent antitumor effects individually or in combination with other therapeutic agents. Based on these two aspects, Kumari et al. (70) synthesized curcumin-supported selenium nanoparticles (SeCurNPs) to achieve an enhanced therapeutic effect. They found that the therapeutic effect of SeCurNPs on colorectal cancer cells (HCT116) was mainly attributable to increased levels of autophagy and apoptosis as autophagy-associated protein (LC3B-II) and proapoptotic protein (Bax) were significantly upregulated and anti-apoptotic protein (Bcl-2) and cytochrome C (Cyt C) were downregulated (70). These results indicated the ability of SeCurNPs for tumor treatment by increasing cancer cells autophagy. In the following work, they further synthesized CD44-targeted DOX loaded nanoparticles (PSHA-DOXNPs) and evaluated their anticancer efficacy in combination with curcumin loaded selenium nanoparticles (Se-Cur NPs) (71). Combination of these nanoparticles (NPs) increased ROS level, decreased mitochondrial membrane potential, induced cell cycle arrest and apoptosis in HCT116 cells, and also enhanced the autophagy of these cancer cells. These results also demonstrated the potential of Se NPs to work with other nanoparticles for synergetic anticancer treatment by regulating autophagy.

Huang et al. (72) synthesized Pleurotus tuber-regium (PTR)-conjugated Se NPs (PTR-Se NPs) and investigated its application in colorectal cancer. They found that PTR-Se NPs can trigger intracellular G2/M phase arrest and promote HCT 116 cell death by activating autophagy through upregulation of intracellular Beclin 1. In addition, they also found that autophagy plays an important role in the apoptotic promotion and induction of cell death by PTR-Se NPs, which can promote the release of pro-apoptotic proteins Bax and Bak and initiate mitochondria-mediated apoptosis (72). This study confirmed the high efficacy of PTR-Se NPs in the treatment of colorectal cancer and found that autophagy plays an important role in promoting apoptosis to induce cancer cell death. In same specific situation, Se NPs could both be autophagy activators and inhibitors. Cui et al. (39) synthesized laminarin polysaccharides (LP) decorated selenium nanoparticles (LP-Se NPs) and found that these nanoparticles could induce mitochondria-mediated apoptosis by upregulating Bax expression, cutting caspase-9, and downregulating Bcl-2 expression. More interestingly, LP-Se NPs can induce early autophagy activation but block late autophagy in these cancer cells. The inhibition of autophagy in the late stage could lead to the failure of damaged organelles to be cleared, which could further aggravate cell apoptosis. The results suggest that LP-Se NPs play a cytotoxic role by inhibiting autophagy and promoting apoptosis.

Biomimetic materials are often capable of subtly affecting tissue development, and regeneration, and are also expected to mediate tumor suppression due to their high similarity to natural tissues. Li et al. (73) prepared hierarchically constructed bone-mimetic selenium-doped hydroxyapatite nanoparticles (B-SeHANs), which recapitulated the uniaxially oriented hierarchical structure of bone HA and could potentially play a dual role in the postoperative treatment of bone tumors *via* the chemotherapy from selenium and the promotion of bone repair by hydroxyapatite. In this work, they found that B-SeHANs could induce excess ROS production and promote autophagy in human MNNG/HOS osteosarcoma cells by activating ROS-mediated JNK pathway and inhibiting Akt/mTOR pathway. This work introduces a viable strategy for the development, evaluation and fundamental study of biomimetic selenium nanoparticles to inhibit tumor growth. Huang et al. (74) proposed dual-targeted modified selenium nanoparticles (u/A-Se NPs) as a biocompatible tumor chemotherapeutic drug, which was proved to promote ROS accumulation and autophagosome formation for synergistic HepG2 cell death. These results collectively suggested that Se NPs might be served as a novel anti-tumor agent due to their ability to promote cancer cell autophagy, which is closely related to their ability to induce high ROS levels.

Selenite, a touted cancer chemopreventive agent, has multiple mechanisms of cytotoxicity in cancer cells that are thought to be induced by selenite metabolites. Bao et al. (75) found that the intracellular metabolism of selenite can produce endogenous Se NPs in cancer cells, and its chelation with heat shock protein 90 can reduce the expression of LC3-II, showing the ability of these endogenous Se NPs for the inhibition of autophagy. In addition, Se NPs can also induce glycolytic inhibition and glycolytic-dependent mitochondrial dysfunction, suggesting that endogenous Se NPs may be the main cause of selenite-induced cytotoxicity. Although the exact mechanisms for how these endogenous Se NPs inhibit cancer cell autophagy remain to be further investigated, this work also introduces the potential for cancer cell inhibition by manipulating the endogenous Se NPs sysnthesis.

**FIGURE 2:**
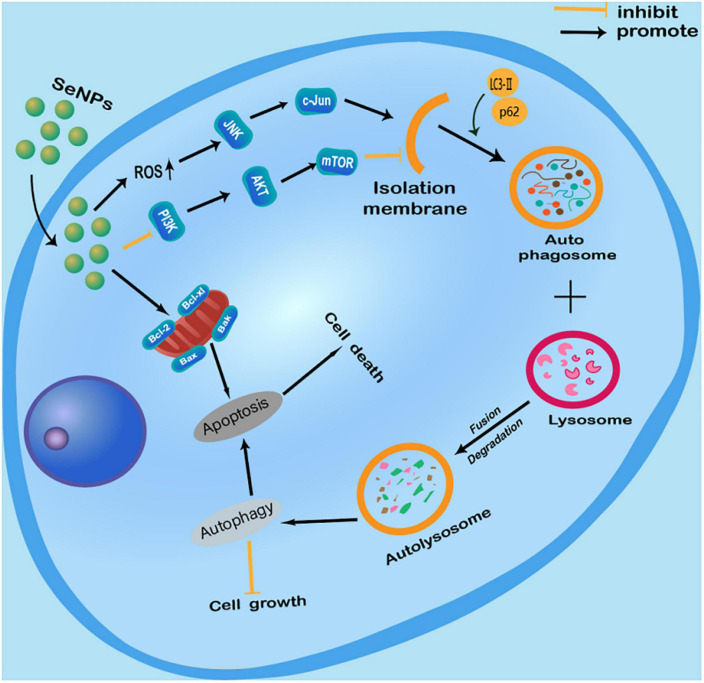
Anticancer effects of selenium nanoparticles (Se NPs) by promoting cancer cell autophagy through the regulation of reactive oxygen species (ROS)-mediated c-Jun N-terminal kinase pathway and PI3K/Akt/mTOR pathway.

We have summarized the current knowledge of how Se NPs inhibit cancer cell growth or induce cancer cell death by promoting cancer cell autophagy (Figure 2). Se NPs are capable of activating ROS-mediated JNK pathway and inhibiting PI3K/Akt/mTOR pathway. These autophagy induction properties of Se NPs could further promote cancer cell apoptosis, which finally results in cancer cell death. Moreover, the ability of Se NPs to promote cancer cell autophagy by activating ROS-mediated JNK pathway and inhibiting PI3K/Akt/mTOR pathway might also be combined with current chemotherapy method for synergetic anticancer treatment.

## Mechanisms for Se NPs regulated autophagy in infectious diseases

Except for the ability of Se NPs to inhibit cancer cells by regulating autophagy, our previous work also indicated the potentials of Se NPs to inhibit intracellular pathogens. Using some immune escaping mechanisms, such as their ability to inhibit autophagy and apoptosis of the host cells, Mycobacterium tuberculosis (Mtb) can escape from the immunological killings of host cells. We previously prepared a kind of Se NPs modified with mannose and encapsulated with isoniazide (Ison@Man-Se NPs) for synergistic drug-induced and phagolysosomal destruction of Mtb. Except for the ability of Ison@Man-Se NPs to promote Mtb-lysosome fusion, apoptosis and M1 polarization in Mtb infected macrophages, we also found that Ison@Man-Se NPs could promote autophagy of Mtb infected macrophages (76). The obtained results also indicated that Se NPs promoted autophagy might be associated with PI3K/Akt/mTOR pathway. And the formation of autophagosomes in the Mtb infected macrophages could finally promote the destruction of Mtb for synergistic intracellular Mtb killings with the anti-tuberculosis drugs. These results indicated that Se NPs, with the ability to promote autophagy of Mtb infected macrophages, could also serve as a kind of novel anti-tuberculosis agents for anti-tuberculosis strategy development. Moreover, these results also indicate the ability of Se NPs for intracellular pathogen clearance by regulating autophagy, which therefore can be served as potential anti-infectious agents.

## Mechanisms for Se NPs regulated autophagy in other diseases

Rheumatoid arthritis (RA), a chronic autoimmune disease, is still lack of effective treatments. Liu et al. (77) prepared Se NPs-PEG-RGD@Ru by modifying selenium nanoparticles with PEG, RGD, and Ru, to target the abundant neovascular network of inflammatory sites, which increased NO production to activate autophagy by modulating signaling AMPK and mTOR pathways and inhibiting the activity of NF-κB-p65 to modulate the levels of inflammatory cytokines in Human Umbilical Vein Endothelial Cells (HUVECs). These unexpected results provided an effective strategy for the target treatment of RA based on the ability of Se NPs to promote autophagy of HUVECs.

Adolescence, a period of intense development, is accompanied by important physiological endocrine, and neurodevelopmental changes. Obesity, insulin resistance, and anorexia nervosa are particularly rising during this period. Ojeda et al. (78) firstly demonstrated *in vivo* that low selenite supplementation could promote adipogenesis through the insulin signaling pathway and lipocalin2 regulation, while low Se NPs administration could prevent fat storage in white adipose tissue and alleviate inflammation by reducing insulin signaling pathway and FOXO3A autophagy. These results provided data for dietary approaches to prevent obesity and/or anorexia during adolescence using Se NPs as autophagy regulators and also highlighted the potential roles of Se NPs for the prevention of adipocyte differentiation.

Se NPs may have a potential role in treating dermal disorders due to their wide therapeutic properties, but there is a need to evaluate their toxicity in keratinocytes. Thus, Kirwale et al. (79) synthesized Se NPs and tested their effects on keratinocytes, which indicated that Se NPs could promote autophagy by inducing AMPK phosphorylation and acidic lysosome formation, which finally enhanced the apoptosis of keratinocytes. These results indicated that Se NPs could induce the oxidative stress and autophagy mediated apoptotic cell death in human keratinocytes cells, which reminds the toxicity issues of Se NPs for further therapeutic application against dermal disorders.

Moreover, Se NPs can also show different protection effects by regulating autophagy. Yan et al. (80) found that Se NPs synthesized by Lactobacillus casei ATCC 393 could attenuate the H_2_O_2_-induced intestinal epithelial barrier dysfunction and ROS overproduction, as well as alleviate the adenosine triphosphate (ATP) level and the mitochondrial membrane potential (MMP) decrease. In addition, these Se NPs inhibited H_2_O_2_-induced phosphorylation of the mammalian target of rapamycin (m-TOR), and the increased expression levels of UNC-51-like kinase 1 (ULK1), light chain 3 (LC3)-II/LC3-I, PTEN-induced kinase 1 (PINK1), and Parkin proteins (80). However, the reasons for the simultaneous increased expression of m-TOR and LC3-II are not well-investigated. It has been widely known that m-TOR can also promote necrosis (81), so we speculate that H_2_O_2_ may induce autophagy and cell necrosis simultaneously, and some unknown signaling events involved in necrosis may promote the expression of m-TOR. In conclusion, these results suggested that these Se NPs can effectively alleviate H_2_O_2_-induced intestinal epithelial barrier dysfunction by regulating mTOR/PINK1-mediated autophagy (80). The same group also found that Se NPs can inhibit protein kinase R-like endoplasmic reticulum kinase (PERK), eukaryotic initiation factor 2 (eIF2α), and the expression levels of CHOP and p-PERK activating transcription factor 4 (ATF4) to inhibit endoplasmic reticulum stress (ERS) in intestinal epithelial cells exposed to H_2_O_2_ (82). And Se NPs were found to regulate endoplasmic reticulum stress-mediated mitophagy by inhibiting the AMPK/mTOR/PINK1 signaling pathway, thereby reducing intestinal epithelial barrier damage (82).

**FIGURE 3:**
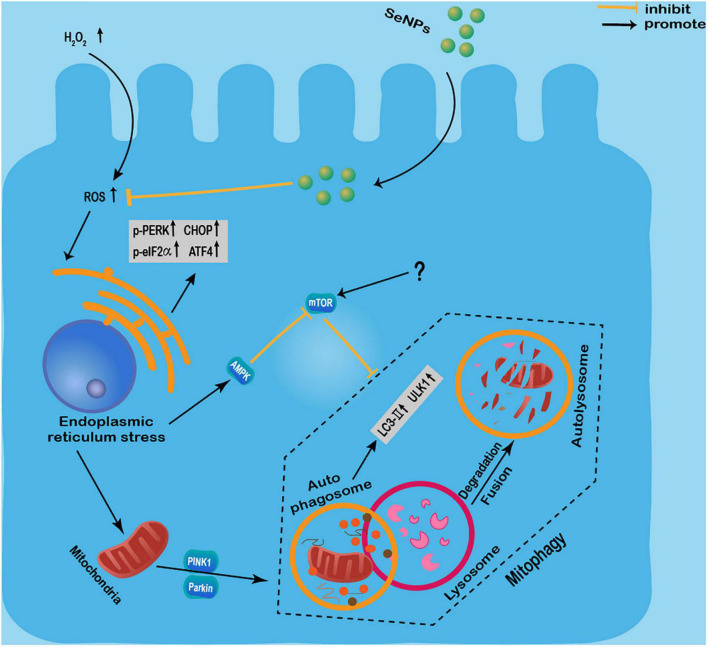
Protective effects of selenium nanoparticles (Se NPs) regulated autophagy against H_2_O_2_-induced cell dysfunction by regulating mTOR/PINK1-mediated pathway, PERK/eIF2α/ATF4 and AMPK/mTOR/PINK1 signaling pathway.

These results suggested the protective effects of Se NPs regulated autophagy against H_2_O_2_-induced cell dysfunction by regulating mTOR/PINK1-mediated pathway, PERK/eIF2α/ATF4 and AMPK/mTOR/PINK1 signaling pathway (Figure 3). Although more works are still needed to further elucidate the exact mechanisms involved in their protective effects, the protective roles of Se NPs in different disease models by regulating autophagy have strongly suggested the potential uses of Se NPs as novel protective agents.

## Potentials of Se NPs for disease treatment through autophagy modulation

Se NPs have attracted widespread attentions in biomedical field due to their unique biological, physical and chemical properties. By the modulation of autophagy, Se NPs exhibit excellent biological activities that can directly contribute to the treatment of cancer, infection and other diseases, which are expected to facilitate the development of novel therapeutics.

Cancer, one of the greatest threats to human health, is causing very high mortality rate worldwide that dramatically threatens human lives (83–85). Currently, the main treatment for cancer is chemotherapy, however, while chemotherapy kills cancer cells, it can also damage normal cells and cause serious side effects that also injure the human health (86–88). Se NPs, a potential anti-cancer agent, can not only directly kill cancer cells, but can also enhance the targeting effects of current chemotherapeutics and achieve synergetic anti-cancer therapy. For instance, adriamycin could be loaded onto Se NPs to achieve enhanced cellular uptake of adriamycin, which therefore resulted in greater anti-cervical cancer activity (89). By promoting the autophagy and apoptosis, Se NPs loaded with curcumin could kill the cancer cells more effectively and inhibit the tumor growth in tumor-bearing mice with prolonged survival time (70). In addition, the combination of curcumin loaded Se NPs and doxorubicin loaded Se NPs can target multiple molecular targets, which is beneficial to enhance anticancer effect (71).

Drug resistance of cancer is a widely-known issue that makes cancer cells to be tolerant to pharmaceutical treatment, which results in the more and more difficult therapy of cancer. Se NPs can also reduce the resistance of tumor cells to chemotherapy drugs by regulating autophagy. It has been shown that Se NPs modified with kelp polysaccharides can inhibit autophagy in HepG-2 cells by reducing the fusion of autophagosomes with lysosomes or decreasing the enzymatic activity of lysosomes, which is beneficial in reducing the resistance of tumor cells to chemotherapeutic agents *in vitro* (39).

Se NPs can also be combined with radiation therapy to increase the autophagy of breast cancer cells as a kind of novel radiosensitizer. For example, Chen et al. (90) reported that Se NPs could reinforce the toxic effects of irradiation, lead to a higher mortality rate than either treatment used alone, induce cell cycle arrest and the activation of autophagy, and increase both endogenous and irradiation-induced reactive oxygen species formation for enhanced radiation therapy efficiency. PEG-modified Se NPs can synergistically inhibit tumor cell growth by DNA fragmentation and caspase-3 activation, which similarly shows radiosensitization to X-rays (91).

Infectious diseases, caused by pathogens infections such as bacteria, fungi and viruses, remain a serious diseases that threaten public health (92, 93). Plenty of works have indicated the ability of selenium compounds to kill intracellular pathogens by regulating the autophagy of pathogen infected host cells (22, 24). The antibacterial and antiviral activities of selenium compounds through modulation of autophagy indicate the potential ability of Se NPs for anti-infection treatments. It has been shown that macrophage targeted Man-Se NPs can kill Mycobacterium tuberculosis (Mtb) by promoting macrophage autophagy (76). This well-designed Se NPs can increase the expression of LC3B-II protein in BCG-infected THP-1 cells by regulating of PI3K/mTOR/AKT pathway, inducing autophagosome formation and promoting the fusion of Mtb into autolysosomes for more effective Mtb killing (76).

Diabetes is a chronic (long-lasting) health condition that affects how your body turns food into energy (94). It has been shown that Se NPs can alleviate the symptoms of weight loss, lower blood glucose levels and improve antioxidant status in diabetic mice (95). Diabetic nephropathy during pregnancy in diabetic female rats can also be alleviated by supplementation with Se NPs (96). These results suggest the promising application of Se NPs for anti-diabetic therapy. Due to critical roles of autophagy in diabetes development (97), we believe that more attentions would be paid into the effects of Se NPs on autophagy regulation for diabetes treatment.

Renal injury is a non-negligible issue for chemotherapy as lots of chemotherapeutic drugs would lead to inevitable renal toxicity (98). The intriguing relationships between Se physiology and several derangements and comorbidities associated with acute and chronic kidney disease have been well-demonstrated in recent years (99). Se NPs have also been proved to efficiently reduce renal tissue injury and regulate the expression pattern of aldose reductase in the diabetic-nephropathy rat model (100), indicating the potentials of Se NPs to relieve renal injury. In addition, Se NPs can also improve gentamicin-induced kidney damage by inhibiting oxidative damage, inflammation and apoptosis-induced by autophagy (101), which introduced a good option of Se NPs as an adjunctive treatment to reduce its side effects for kidney damage.

## Conclusion and perspectives

Autophagy is an evolutionarily conserved process that mediates the degradation of long-lived proteins and damaged organelles in response to a variety of stressful stimulus, including starvation, oxidative stress, and viral infection (1, 102, 103). During autophagy, the cytoplasmic fraction is isolated in autophagosomes, which eventually fuse with lysosomal compartments for overall degradation (104, 105). Se is an essential trace element in animals, and has been proved to show important biological function in anti-oxidative stress, anti-tumor and improving the immunity of the body. Different forms Se (including sodium selenite, selenoprotein, and selenium-rich yeast) act in different ways in the human body, and Se NPs have attracted increasing of attention in recent years due to their attractive biological activities (26, 106, 107). Here, we summarized the current research progress on the ability and how could Se NPs induce tumor cell death, reduce drug toxicity, modulate inflammatory responses, resist pathogenic bacterial infections, treat Alzheimer’s disease as well as other diseased conditions by regulating autophagy.

Although Se has showed attractive biological activities, selenium compounds are widely restricted by their high toxicity and poor targeting. Selenium compounds, such as sodium selenite, have strong anticancer effects only at high doses, but the toxicity at high doses just provided a weak gap between beneficial and toxic effects. Therefore, toxicity has been one of the key issues in the development of selenium-based anticancer drugs. Se NPs, a new type of monomeric selenium, have indicated strong biological activities, such as low toxicity, high bioavailability, regulation of selenoprotein functions, scavenging of free radicals, protection of oxidative DNA damage, and strong anti-cancer effects compared to inorganic or organic selenium compound (29, 108–110). In addition, taking the advantages of good biocompatibility, high loading rate, low toxicity, easy synthesis and easy storage, Se NPs can also serve as drug carriers to improve the solubility and stability with prolonged cycle time, thus increasing drug efficiency (33, 111, 112).

More importantly, increasing studies are suggesting that Se NPs can serve as a kind of novel autophagy regulators, which might provide the potential use of Se NPs against different diseased conditions. In recent years, the ability of Se NPs for autophagy regulation has been proved to show strong potentials in some impotent diseases, including cancer, tuberculosis, rheumatoid arthritis, adolescent obesity or anorexia, skin diseases, diabetes and kidney injury. The attractive bioactivity of Se NPs for autophagy modulation not only suggests Se NPs for disease treatment alone (78, 79), but also introduces the possibilities to combine Se NPs and current chemotherapeutics to achieve enhanced efficiency (70, 71), which would be beneficial in facilitating the development of new therapies.

However, there are still lots of critical issues for the further clinical application of Se NPs by regulating autophagy. Firstly, although some works have indicated the potential mechanisms of Se NPs induced autophagy, most of these works just provided some phenotype results without depth investigations (68, 75, 78). To further confirm the detailed mechanisms of Se NPs induced autophagy, knockout/knockdown experiments in cell and mice models are needed. These works would further explain whether these potential pathways are critical for the control of Se NPs induced autophagy. Secondly, the relations between autophagy and apoptosis are also needed to be further investigated in Se NPs induced autophagy, as most of the results indicated that Se NPs could both induce autophagy and apoptosis (63, 67, 73).

Thirdly, the toxicity issue of Se NPs should be considered as one of the most important issues for the further application of Se NPs. Although some works have indicated that Se NPs could inhibit cancer cells selectively without significant inhibition effects on normal cells (63, 89), the detailed mechanisms for these selectivity are still unknown. And there are still few studies that specifically focus on the systemic toxicity of long-term Se NPs treatment *in vivo*. Although Se NPs have been claimed to show relatively lower toxicity and higher degradability, the degradation and metabolism processes of Se NPs *in vivo* have not been well-explored. More concerns should be paid to the safety issues of Se NPs to investigate how they uptake, metabolize, degrade and eliminate in animal models, as well as how they interact with normal tissues and cells *in vivo* and *in vitro*, on account of excessive Se causing toxic symptoms.

Overall, taking the advantages of their advanced ability to regulate autophagy, Se NPs have demonstrated attractive potentials for the treatment of different diseases. And these autophagy regulation effects of Se NPs can be served as an adjuvant treatment to boost the efficiency of current therapeutics, such as chemotherapy, radiotherapy and immunotherapy. With the increased understanding of their functions and mechanisms, especially their metabolism, degradation and long-term safety *in vivo*, we believe that Se NPs could be applied as kind of novel autophagy regulators, which might provide new possibilities to benefit the clinical therapeutics against some important diseases.

## Author contributions

DC and HL drafted this manuscript. YM, SF, YuH, TZ, WL, and YiH helped to revise the manuscript. YR, J-FX, and JP helped to revise the manuscript and were responsible for leading this work. All authors contributed to the article and approved the submitted version.
